# Supplementary material for: Nominal elastic modulus assessment in 3D-printed components under varying printing parameters using Bayesian methods and random forest surrogate modeling
Source: PLoS One. 2025 Dec 5;20(12):e0338204. doi: 10.1371/journal.pone.0338204 (PMC12680227; doi:10.1371/journal.pone.0338204)
Supplement: Attachment — Submitted filename: 2025.09.04 Response to reviewers.docx [file pone.0338204.s004.docx]

**Additional Editor Comments:**

The manuscript must be revised based on the reviewers’ comments plus the following issues,

1) A separated file must be provided for the authors’ answers to the comments, one by one. Moreover, all changes must be yellow-colored highlighted sentences in the revised article. The track changes condition is not suggested.

Answer: We have provided a separate point-by-point response file. All changes in the revised manuscript are highlighted in yellow, and we did not use “Track Changes.”

2) No abbreviations should be used in the keywords. They must also be found in the abstract or the title.
Answer: We have removed all abbreviations from the keywords and ensured the corresponding terms appear in the Abstract and/or Title as required.

3) The introduction is lengthy. Just 3-page is enough.
Answer: The Introduction has been condensed and now fits within three pages.

4) All formulations need references, unless they were extracted or introduced by the authors.
Answer: All equations are now properly cited.

5) The scale bar must be provided for macroscopic and microscopic images.
Answer: Scale bars have been added for the macroscopic images; no micro-scale images are included in this manuscript.

6) The figure title is too brief and nothings could be understood from the figure title.

Answer: Several figure captions have been expanded so that the figures can be understood from the captions alone.

7) The structure is confusing. The main text must include the introduction, the research method, the results and discussion, conclusions, and references.

Answer: We reorganized the manuscript to follow the required structure (Introduction, Methods, Results and Discussion, Conclusions, and References) and added a dedicated Discussion section.

8) The elastic module needs references in Table 3. If measured by the authors, more details must be provided with the repeatability of testing.
Answer: All elastic-modulus values in Table 3 are now referenced.

9) “Conclusion” must be changed to “Conclusions”. Then, the conclusion section should be rewritten one by one, in bullets, to show the novelty. In addition, it is too lengthy and it should be shortened.

Answer: We have changed “Conclusion” to “Conclusions,” rewrote the section point by point to highlight the novelty, and shortened it accordingly. Please see the revised manuscript.

10) All used material properties for finite element modeling must be mentioned with references.
Answer: All material properties used for the finite-element modeling are now explicitly listed and referenced, and additional modeling details have been provided.

11) The discussion is poor and it must be improved. They must be compared to other results of other similar articles.

Answer: The Discussion has been substantially strengthened, including comparisons with related studies.

12) References should be updated based on recent articles, published in 2015-2025. Moreover, it should be extended to at least 40 articles for a proper discussion.

Answer: The reference list has been updated to focus on 2015–2025 publications and expanded to 40 items.

**Reviewer #1: The paper is of high quality, and I appreciate the solid methodology. However, some revisions are required before publication:**
1. Introduction:
The introduction is well written, and the authors clearly present the motivation and novelty of their work. However, two improvements are suggested:
• Please avoid citing more than three references in a single sentence.
• The overall length of the paper is quite high, which makes it difficult to read in full. Consider condensing some sections to improve readability.

**RESPONSE**: We appreciate your constructive suggestions. The Introduction has been streamlined by condensing unnecessary content and refining the presentation of references. These revisions can be found in the updated manuscript.

**RELATED REVISIONS**

**Page 1**

1.	Introduction

3D printing has significantly advanced the industrial lightweighting process through its capabilities in free design, topological optimization, and the formation of complex structures [1]. Due to its highly adaptable manufacturing capabilities and ease of use, 3D printing has found widespread application in the rapid prototyping of complex geometries in metals, polymers, and fiber-reinforced composites [2]. Plastic filament is melted by heating and then layer by layer deposited through a nozzle, gradually accumulating to form the finalized shape. This technology features advantages such as fast processing speeds, low costs, and simple equipment, making it widely used for the manufacturing of functional and structural components [3].

Fused Deposition Modeling (FDM) is one of the most representative rapid prototyping methods within 3D printing [4]. The FDM process can achieve low-cost preparation of high-performance composite materials while reducing subsequent processing steps and offering good recyclability and reprocess ability. However, the forming accuracy of FDM components is largely determined by the precision of the FDM machine itself, and errors can occur at each stage of the manufacturing process, affecting the final precision [5]. Recently, numerous studies have evaluated the impact of FDM process parameters such as layer thickness [5], nozzle temperature [6], infill density [7], and printing speed [8] on the mechanical properties of manufactured parts. These studies have found that these factors significantly influence the strength of the produced components.

Due to the influence of interlayer adhesion, void defects, and various coupled effects of process parameters during the manufacturing of FDM components, obtaining accurate elastic parameters is essential for subsequent structural safety assessments and optimization designs. Currently, the commonly used methods for characterizing mechanical properties predominantly rely on destructive testing, such as uniaxial tensile tests [9], compression tests [10], shear tests [11], and fatigue tests [12]. However, these methods not only damage the test specimens but also require the preparation of samples that conform to standard dimensions. For components that are limited in volume, scarce in samples, or already formed functional parts, it is often difficult to conduct repeated testing and obtain information regarding the internal anisotropy of the components.

To overcome these limitations, recent years have seen a diverse range of research focused on the calibration of material parameters for 3D printed materials and components. Lesueur et al. [13] utilized numerical modeling to predict the yield strength of porous materials and examined the impact of internal geometries using an elastoplastic model. Luo et al. [14] conducted static and dynamic experiments on 3D printed components made of PLA and PLA-Cu, simulating their stress-strain curves. Somireddy et al. [15] proposed a constitutive model for 3D printing based on tensile test data to perform numerical simulations of elastic material properties. Khosravani [16] conducted a series of static tensile tests to analyze the effects of raster orientation and printing speed on the mechanical properties of materials. Lißner et al. [17] established a framework combining experimental and numerical methods, using experimental data from solid 3D printed compression specimens to calibrate material models, and validated the accuracy of the calibrated models using samples of different geometries. These advancements have greatly facilitated progress in the parameter calibration of 3D printed components. However, some shortcomings remain. For instance, most studies primarily focus on identifying various mechanical parameters from the material level, lacking parameter identification methods based on finite element model updating at the component level to accurately quantify structural mechanical parameters. Moreover, many works inadequately consider the inherent randomness of materials and the uncertainty of testing, resulting in relatively singular identification outcomes.

Similar challenges are also widespread in the fields of civil engineering and aerospace. In these domains, researchers often employ model updating methods for parameter inference and performance prediction, as some critical parameters are difficult to measure directly [18-19]. However, traditional model updating methods typically rely on deterministic assumptions, making it challenging to comprehensively quantify uncertainties in both the model and observations. In recent years, Bayesian model updating methods have gained widespread attention for their ability to systematically integrate prior knowledge with observational data while naturally handling uncertainties, providing a more robust and flexible approach for parameter identification and model updating [22-23].

Manufacturing parts with stable mechanical properties has always been a challenging task in FDM technology; therefore, obtaining accurate material mechanical parameters is particularly important for predicting the mechanical performance of 3D printed structures. Based on this, the present study intends to introduce a Bayesian model updating method based on Markov Chain Monte Carlo (MCMC) sampling, combined with finite element modeling, to achieve precise identification and calibration of elastic moduli for 3D printed materials, thereby enhancing the accuracy and reliability of mechanical performance predictions for 3D printed components. Considering that the Bayesian MCMC method requires multiple runs of complex finite element models, resulting in high computational costs, this study incorporates advanced random forest algorithms from machine learning to replace time-consuming finite element reanalysis, achieving efficient mapping between structural parameters (inputs) and structural responses (outputs) to enhance the reliability and application value of 3D printed material performance predictions.

2. Sample Manufacturing and Material Properties:
Was any repetition in the manufacturing of samples considered? How did you assign a specific elastic modulus to the parts if no replication was performed? Please clarify this point.

**RESPONSE:** Thanks the reviewer for raising this important point. In fact, multiple specimens were manufactured and tested. We observed that even for the same nominal material from different suppliers, slight variations in the measured frequencies existed despite identical printing parameters. However, these variations were considerably smaller than those arising from different infill ratios within the same batch of material. Therefore, we assigned a nominal unified elastic modulus as the initial value for each material type, and subsequently calibrated the actual elastic modulus of each specimen using the measured frequency responses.

3. Elastic Modulus in FEM Model:
A critical point to consider is how the elastic modulus was defined in the FEM model. AM parts produced by the FDM process typically exhibit different elastic properties depending on orientation. By assigning a single elastic modulus, how do you account for anisotropy? I suggest discussing whether the parts were considered isotropic or not, and whether a composite-like modeling approach would have been more appropriate.

**RESPONSE:** Although FDM-printed components generally exhibit anisotropic behavior due to their layer-by-layer deposition process, the present study focuses solely on responses in the vertical bending direction. In this specific orientation, the internal infill is deposited in a uniform and consistent manner across all specimens, such that the structural response can be effectively represented by a single equivalent elastic modulus. This isotropic approximation in the bending direction has also been adopted in prior studies, where the elastic modulus is treated as a macro-scale equivalent parameter rather than a material constant, thereby ensuring that the model remains both computationally efficient and physically meaningful.

4. FEM Simulations:
The FEM simulations are not described with sufficient detail for a scientific paper. Please clarify:
• What software was used?
• What settings were used on software?
• What boundary conditions were applied?
Figure 7 is helpful, but the degrees of freedom in the simulations should also be fully specified.
I recommend following the approach of the following papers, which present their FEM setup clearly with tables and schematic figures: Abaqus: Theoretical and numerical stress analysis in the cam of a medium voltage switchgear vacuum circuit breaker supported by image processing of deformation (see Table 3). COMSOL: Modeling Nonlinear Deformation in Magnetic Polyelectrolyte Hydrogels: A Hybrid FEM-Machine Learning Framework (see Figure 3 and Table 1). Please provide a similar table or figure summarizing your simulation details. In addition, include some representative mode shapes from your frequency analysis (FEM contours) and describe the software process used to extract the natural frequencies. As someone experienced with FEM, I find the current description too vague to replicate your approach.

**RESPONSE**: Thanks for your observation and the references provided. In the original version, we indeed lacked sufficient detail regarding the finite element modeling. In the revision, we clarify that the structural model was developed using the ANSYS software package with the BEAM188 element. A cantilever boundary condition was imposed by fully fixing one end of the beam. The beam was discretized uniformly into 22 segments, each with a length of 10 mm. The cross-section of the beam measured 15 mm × 15 mm. As illustrated in Figure 7, a 6 g sensor mass was added at the free end and another 6 g sensor mass was attached at midspan, both modeled using the MASS21 element. The structural mass was uniformly distributed, and the total mass can be found in Table 1. We carefully studied the references you suggested and revised Figure 7 accordingly, these updates are incorporated in the revised manuscript.

**RELATED REVISIONS**

**Page 15**

As shown in Fig. 5(b), the finite element model of the specimen was established in ANSYS using the BEAM188 element. BEAM188 is a three-dimensional beam element suitable for simulating bending, shear, and torsional deformations. The beam has a square cross-section of 1.5 cm × 1.5 cm, with a total length of 25 cm. The rightmost 3 cm of the beam was clamped to represent the fixture, thereby applying a fixed boundary condition at the nodes of this region. The beam was discretized along its longitudinal axis into 22 elements, each 1 cm in length. The mass of each segment was assigned according to the actual mass values listed in Table 1, assuming a uniform distribution. In addition, the mass of the sensor and its connecting cable (6 g) was incorporated into the initial model as a lumped mass through the MASS21 element, thereby accounting for the extra inertia introduced by the measurement system.

5. Machine Learning Algorithm Choice:
Why was a Decision Tree (DT) chosen for your study? Specifically:
• What was the rationale for using a tree-based model?
• Why were more advanced models such as Random Forest or XGBoost not considered?
In most recent studies, DT alone performs poorly compared to these ensemble methods. While your accuracy appears high, a scientific paper should provide justification for algorithm selection, ideally with comparisons. For reference, see: Shapley additive explanation on machine learning predictions of fatigue lifetimes in piston aluminum alloys under different manufacturing and loading conditions.

**RESPONSE**: We agree with the reviewer’s insightful comment that advanced ensemble models such as Random Forests or XGBoost can offer higher predictive power, especially in scenarios involving high-dimensional inputs or strongly nonlinear relationships. However, in our case the 3D-printed specimens are manufactured with a highly uniform process, so the elastic modulus across different regions can be reasonably assumed to be homogeneous. Accordingly, we treat the elastic modulus of each specimen as a single parameter to be identified. Moreover, since our study is restricted to the elastic range, the input–output relationship between the elastic modulus and the modal responses (natural frequencies and mode shapes) is approximately linear. Under such conditions, a conventional decision tree model is fully sufficient to capture this mapping. In contrast, more advanced models like Random Forests or XGBoost are most beneficial for higher-dimensional or more complex nonlinear problems, and they also involve more elaborate hyperparameter tuning. Considering the research objectives and the need for simplicity and reproducibility, we therefore opted for a decision tree model. We have added a discussion of this rationale in the revised manuscript.

6. ML Training Process:
The training details of the DT model are unclear. Please specify:
• Did you use a standard package or library implementation?
• Were hyperparameters tuned, and if so, how?
• What data splitting strategy was applied (e.g., train/test split, cross-validation)?
These details are essential for reproducibility.

**RESPONSE**: We appreciate the reviewer’s comment. In our study, the random-forest surrogate (RF) was implemented using MATLAB’s built-in fitrensemble function (Method='Bag'), with CART regression trees as base learners. A total of 600 elastic modulus samples were generated within [400,900] MPa by Latin hypercube sampling. For each sample, the finite element (FE) model predicted the first two natural frequencies and two nodal value of the mode shapes. which served as outputs to train two RF regressors. The dataset was divided into 500 training and 100 test samples with a fixed random seed. To improve robustness, we introduced a semi-noise augmentation strategy: 1% multiplicative Gaussian noise was added to the output frequencies of 50% of the training samples, while inputs and test data remained noise-free. For hyperparameters, we used B=300B trees; the maximum depth and minimum leaf size were kept at MATLAB defaults. A small sensitivity analysis showed that increasing the number of trees beyond 300 provided negligible gains, so this value was fixed. Model accuracy was evaluated on the independent test set using the Pearson correlation coefficient (R) and mean squared error (MSE). These additional details have been included in the revised manuscript to improve clarity and reproducibility.

**RELATED REVISIONS**

**Page 19**

**3.4 Finite Element Model Updating**

Considering the notable differences between the measured frequencies and mode shapes and their previously calculated values, the updating of the nominal elastic modulus for the nine 3D-printed components will follow the procedure outlined in Figure 1. The data used for model updating includes the first two measured frequencies and the first two mode shapes from the two measurement points shown in Figure 7. The updating factor is defined as θ=Er/E0 , where E_r_ denotes the actual elastic modulus of the structure and E_0_ is the nominal value. Based on the selected response indices, a RF surrogate model is first constructed. Taking the PLA specimen with a 30% infill ratio as an illustrative example, a total of N=600 elastic modulus samples were generated within [400,900] MPa using Latin hypercube sampling. For each sample, the FE model provided the first two natural frequencies and two nodal values of the mode shapes, which were used as outputs for training four separate RF regressors. The dataset was split into 500 training and 100 test samples with a fixed random seed. To improve robustness to measurement perturbations while preserving unbiased targets, a 1% multiplicative Gaussian noise was applied to the output frequencies of 50% of the training samples (semi-noise augmentation), while inputs and test data remained noise-free. The RF models were implemented with MATLAB’s standard fitrensemble function (Method='Bag'), using B=300 regression trees with bootstrap aggregation of CART base learners. No explicit depth limit was enforced, and the minimum leaf size was kept at the default value; these settings were chosen after a sensitivity analysis indicated negligible gains beyond B≈300B. Model accuracy was evaluated on the independent test set using the Pearson correlation coefficient (R) and mean squared error (MSE).

Figure 8 illustrates the prediction accuracy of the first two natural frequencies for the PLA specimen with a 30% infill ratio. As shown in Figure 8, the frequencies predicted by the RF model are in excellent agreement with those obtained from the FE analysis, confirming the effectiveness of the RF surrogate in approximating the modal responses of this specimen.

|  |  |
| --- | --- |
| (a) First natural frequency | (b) Second natural frequency |

**Figure 8** Prediction of natural frequencies by the RF surrogate model for the PLA specimen with 30% infill ratio.

Subsequently, Eqs. (1)–(5) were employed to construct the Markov chains for the updating factors of each component and to estimate the mean values of their elastic moduli.

7. Conclusions:
The conclusions section needs reorganization:
• Change the section title to Conclusions.
• Begin with a concise statement of the methodology.
• Present the main results in bullet points.
• Highlight the key outputs of the paper with quantitative results.

**RESPONSE**: We thank the reviewer for this valuable suggestion. In the revised manuscript, the section title has been changed from *Conclusion* to *Conclusions*. Moreover, the content of this section has been reorganized to make the key findings more prominent, with the main points presented more clearly and supported by quantitative discussion.

**RELATED REVISIONS**

**Page 25**

**4. Conclusions**

This paper presented and verified an efficient, non-destructive Bayesian framework for parameter identification, which integrates FE models, a RF surrogate model, and probabilistic inference to calibrate the equivalent elastic modulus of FDM 3D-printed components.

**Main results and findings:**

- **Specimen design and testing:** Nine cantilever beam specimens were fabricated using three materials (PLA, PETG, ASA) and three infill densities (5%, 15%, 30%). Operational modal analysis successfully obtained the first two natural frequencies and mode shapes.
- **Surrogate modeling:** A RF surrogate model was trained to approximate the nonlinear relationship between elastic modulus and modal responses, significantly reducing the computational cost of Bayesian MCMC-based updating.
- **Parameter identification:** Bayesian inference combined with RF surrogate model yielded the posterior distributions of equivalent elastic moduli for all specimens.
- **Key quantitative results:**
  (1) PLA exhibited the strongest dependence on infill density, with a 29.2% increase in modulus between 5% and 30% infill.
  (2) Frequency prediction errors of the updated FE model were largely within 3%, and all modal assurance criterion (MAC) values exceeded 0.99, confirming high accuracy and physical consistency.

**Contributions and implications:**

- Demonstrated that the equivalent elastic modulus of FDM components is a macro-scale property dependent on infill density, rather than a fixed intrinsic material constant.
- Provided a non-destructive method for directly calibrating the mechanical performance of printed components at the structural level, eliminating the need for destructive coupon tests.
- The proposed framework offers reliable data for quality control, performance prediction, and design optimization, and is extendable to more complex geometries, other additive manufacturing processes (e.g., SLA, SLS), and additional physical parameters such as damping and anisotropy.
- Future work will explore integration into automated quality inspection and multi-scale modeling to establish digital closed-loops from design and manufacturing to performance verification.

**Reviewer #2: This manuscript presents a novel and robust framework for the non-destructive identification of the nominal elastic modulus in 3D-printed components. The combination of Bayesian inference with a machine learning surrogate model is a powerful approach to overcome computational costs. The work is scientifically sound, well-organized, and has significant practical value for the additive manufacturing community. I recommend Major Revision to address the following points which will substantially strengthen the paper before it is acceptable for publication.**

1 The entire study is predicated on the assumption that the printed infill density (5%, 15%, 30%) is accurately reflected in the final specimens. This is a critical parameter, yet no evidence is provided to verify the actual printed internal geometry. The authors must include SEM (Scanning Electron Microscopy) or optical micrograph images of cross-sections of the printed specimens (one for each infill density). This is essential to confirm that the internal void structure and infill pattern correspond to the designed densities in the slicing software. Without this validation, the correlation between infill density and elastic modulus remains an unverified assumption.

**RESPONSE**: We sincerely thank the reviewer for this thoughtful suggestion. Indeed, Figure 3 of the manuscript already provides visual evidence of the internal structures at the three infill ratios, although only at a macroscopic level. We fully agree that SEM-level cross-sectional images would provide stronger validation of the internal void morphology and infill pattern. Unfortunately, due to current experimental limitations, we were not able to perform SEM imaging for this study.

We would like to emphasize that the focus of the present work is not on the microscopic material characteristics but rather on the structural-scale effective elastic modulus in the vertical bending direction. This equivalent modulus serves as a key parameter for predicting the macroscopic mechanical performance of FDM-printed components, and our proposed Bayesian-based method is designed as a nondestructive tool for inferring such effective structural properties. Nevertheless, we appreciate the reviewer’s insightful recommendation and will incorporate SEM or optical micrograph validation of infill geometry in our future work.

2 Figures 8, 9, and the one referred to as "Model Shape of PLA" (Fig. 6?) appear to be standard Excel charts. These are not suitable for a high-quality scientific publication. All graphs and charts (especially the bar chart of elastic moduli and the frequency error comparison) must be recreated using professional graphing software such as Origin, Python (Matplotlib/Seaborn), or MATLAB. These tools produce publication-quality figures with proper resolution, font sizes, axis labels, and styling. Please do not submit images of Excel charts.

**RESPONSE**: We have revised Figures 6, 8, and 9. All plots have been re-generated using Python for improved clarity and reproducibility. The updated figures and corresponding descriptions can be found in the revised manuscript.

3 The section on the Random Forest surrogate model (Section 2.2) is well-explained theoretically but lacks critical practical details. Please specify:
♣ The size of the training dataset (N) generated via Latin Hypercube Sampling.
♣ The key hyperparameters of the Random Forest model used (e.g., number of trees B, maximum depth, minimum samples per leaf). Was any hyperparameter tuning performed (e.g., via cross-validation)?

**RESPONSE**: We appreciate the reviewer’s comment. In our study, the random-forest surrogate (RF) was implemented using MATLAB’s built-in fitrensemble function (Method='Bag'), with CART regression trees as base learners. A total of 600 elastic modulus samples were generated within [400,900] MPa by Latin hypercube sampling. For each sample, the finite element (FE) model predicted the first two natural frequencies and two nodal value of the mode shapes. which served as outputs to train two RF regressors. The dataset was divided into 500 training and 100 test samples with a fixed random seed. To improve robustness, we introduced a semi-noise augmentation strategy: 1% multiplicative Gaussian noise was added to the output frequencies of 50% of the training samples, while inputs and test data remained noise-free. For hyperparameters, we used B=300B trees; the maximum depth and minimum leaf size were kept at MATLAB defaults. A small sensitivity analysis showed that increasing the number of trees beyond 300 provided negligible gains, so this value was fixed. Model accuracy was evaluated on the independent test set using the Pearson correlation coefficient (R) and mean squared error (MSE). These additional details have been included in the revised manuscript to improve clarity and reproducibility.

**RELATED REVISIONS**

**Page 19**

**3.4 Finite Element Model Updating**

Considering the notable differences between the measured frequencies and mode shapes and their previously calculated values, the updating of the nominal elastic modulus for the nine 3D-printed components will follow the procedure outlined in Figure 1. The data used for model updating includes the first two measured frequencies and the first two mode shapes from the two measurement points shown in Figure 7. The updating factor is defined as θ=Er/E0 , where E_r_ denotes the actual elastic modulus of the structure and E_0_ is the nominal value. Based on the selected response indices, a RF surrogate model is first constructed. Taking the PLA specimen with a 30% infill ratio as an illustrative example, a total of N=600 elastic modulus samples were generated within [400,900] MPa using Latin hypercube sampling. For each sample, the FE model provided the first two natural frequencies and two nodal values of the mode shapes, which were used as outputs for training four separate RF regressors. The dataset was split into 500 training and 100 test samples with a fixed random seed. To improve robustness to measurement perturbations while preserving unbiased targets, a 1% multiplicative Gaussian noise was applied to the output frequencies of 50% of the training samples (semi-noise augmentation), while inputs and test data remained noise-free. The RF models were implemented with MATLAB’s standard fitrensemble function (Method='Bag'), using B=300 regression trees with bootstrap aggregation of CART base learners. No explicit depth limit was enforced, and the minimum leaf size was kept at the default value; these settings were chosen after a sensitivity analysis indicated negligible gains beyond B≈300B. Model accuracy was evaluated on the independent test set using the Pearson correlation coefficient (R) and mean squared error (MSE).

Figure 8 illustrates the prediction accuracy of the first two natural frequencies for the PLA specimen with a 30% infill ratio. As shown in Figure 8, the frequencies predicted by the RF model are in excellent agreement with those obtained from the FE analysis, confirming the effectiveness of the RF surrogate in approximating the modal responses of this specimen.

|  |  |
| --- | --- |
| (a) First natural frequency | (b) Second natural frequency |

**Figure 8** Prediction of natural frequencies by the RF surrogate model for the PLA specimen with 30% infill ratio.

Subsequently, Eqs. (1)–(5) were employed to construct the Markov chains for the updating factors of each component and to estimate the mean values of their elastic moduli.

4 The results show a surprising trend: higher infill density leads to a decrease in measured frequency (Table 2), which contradicts the intuitive expectation that a denser, stiffer part should have a higher natural frequency. The authors correctly identify that this is due to a dominant mass increase effect, but this counter-intuitive result needs a much stronger and more detailed discussion. Explain the stiffness-mass relationship in the context of natural frequency (ω ∝ √(k/m)) and why the mass increase outweighs the stiffness increase in these specific FDM structures.

**RESPONSE**: Thanks the reviewer for this insightful comment. We have expanded the discussion in Section 3.3 to explicitly explain the stiffness–mass trade-off （ω ∝ √(k/m)） and quantitatively show that the increase in mass with higher infill outweighs the stiffness gain, which accounts for the observed decrease in measured frequencies.

**RELATED REVISIONS**

**Page 16**

**3.3 Modal Testing Results**

The results in Table 2 show that the calculated first two modal frequencies of components fabricated from three materials (PLA, PETG, ASA) decrease markedly with increasing infill density. This behavior can be explained using the dynamic relation for natural frequencies,, where *E* is the elastic modulus and *M* is the structural mass（see Table 1）. Since the nominal elastic modulus used in the calculations is kept constant for all specimens, while the structural mass increases with infill density, the ratio *E*/*M* decreases, leading to lower calculated frequencies. Therefore, the trend of the calculated frequencies is negatively correlated with the variation in infill density.

On the other hand, the experimental modal results reveal that the measured natural frequencies of the three materials change only slightly with different infill densities. This is an interesting phenomenon, because intuitively, higher infill ratios should increase the elastic modulus or stiffness of the structure. However, the experimental results suggest otherwise. From the same relation,, it can be seen that as the infill ratio increases, both the elastic modulus and the structural mass increase. When the rates of increase are comparable, the ratio *E*/*M* remains nearly constant, and thus the natural frequency does not vary significantly. This conjecture will be further examined and confirmed in subsequent sections based on the identified elastic moduli.

Comparing the initially calculated frequencies with the experimentally measured values for the three materials (PLA, PETG, and ASA) reveals the frequency errors corresponding to the three infill densities (5%, 15%, and 30%). The maximum error in the first-order frequency is −15.44%, while that in the second-order frequency is −10.6%. These results indicate that the elastic modulus assumed in the initial FE model is lower than that of the actual specimens, thus requiring model updating.

**Page 21**

To clearly present the updated elastic modulus results, Figure 9 provides the bar chart of the elastic moduli for the nine components. As shown in Figure 9, when compared at the same infill ratio, PLA consistently exhibits the highest modulus and PETG the lowest. At 5% infill, the modulus of PLA is 29.2% higher than that of PETG; at 15% infill, the difference remains 23.2%; and at 30% infill, PLA exceeds PETG by 32.3%. Furthermore, by comparing the relative increases in elastic modulus and mass (with respect to the 5% infill case), we identified the underlying reason why the measured frequencies of all specimens change only slightly with infill. Taking PLA as an example, when the infill ratio increases from 5% to 30%, the total mass—including the 12 g sensor—shows a growth rate of 40.94%, whereas the elastic modulus increases by only 23.8%. According to the dynamic relation, this corresponds to an estimated frequency reduction of approximately −8.6%, which well explains why the measured natural frequencies decrease slightly with increasing infill ratio. It should be noted, however, that a more refined prediction would require accounting for the fact that the sensor mass is positioned at the mid-span and free end of the cantilever, which influences the distribution of mass and thus the measured frequencies.

5 The error for the ASA 30% sample is notably higher (-7.15% and 6.248%). The authors should briefly speculate on the potential reasons for this outlier. Could it be related to material-specific printing artifacts, model inaccuracies, or measurement noise?

**RESPONSE**: We thank the reviewer for this observation. The larger deviation in the ASA specimen at 30% infill can reasonably be attributed to measurement noise and small variations in clamping boundary conditions. The additional mass of the sensor and slight changes in clamping stiffness are sufficient to explain the observed frequency shift. We have added this explanation in the revised manuscript.

**RELATED REVISIONS**

**Page 22**

The error for ASA at a 30% infill rate is slightly larger, with a first-order frequency error of -7.15% and a second-order frequency error of 6.248%, the larger deviation can be reasonably attributed to measurement noise and slight differences in boundary conditions at the clamp. The additional mass of the sensor and small variations in clamping stiffness are sufficient to explain the observed frequency shift.

6 Table 3: The table lists an "Updating factor" and "Elastic Modulus". The definition and purpose of the "Updating factor" are not immediately clear from the text. Please define it in the table caption or the main text.

**RESPONSE**: We thank the reviewer for pointing this out. In the revised manuscript, we have clarified that the updating factor is defined as θ=Er/E0 , where Er is the identified elastic modulus and E0 is the nominal value. This definition has been added to the text and table caption.

**RELATED REVISIONS**

**Page 19**

The updating factor is defined as , where denotes the actual elastic modulus of the structure and E_0_ is the nominal value.
