# Supplementary material for: Nominal elastic modulus assessment in 3D-printed components under varying printing parameters using Bayesian methods and random forest surrogate modeling
Source: PLoS One. 2025 Dec 5;20(12):e0338204. doi: 10.1371/journal.pone.0338204 (PMC12680227; doi:10.1371/journal.pone.0338204)
Supplement: Attachment — Submitted filename: renamed_25bea.docx [file pone.0338204.s005.docx]

**Additional Editor Comments:**

The manuscript must be revised based on the reviewers’ comments plus the following issues,

**1)** **Still, there are some formulations without references.**

Answer: Thanks for pointing this out. We have carefully checked every references and added authoritative citations at the point of introduction. The new modification have been marked in red in the revised manuscript for clarity:

Moreover, many works inadequately consider the inherent randomness of materials and the uncertainty of testing, resulting in relatively singular identification outcomes[18].

In recent years, Bayesian model updating methods have gained widespread attention for their ability to systematically integrate prior knowledge with observational data while naturally handling uncertainties, providing a more robust and flexible approach for parameter identification and model updating [21-23].

Considering that the Bayesian MCMC method requires multiple runs of complex finite element models, resulting in high computational costs, this study incorporates advanced random forest algorithms[24-25] from machine learning to replace time-consuming finite element reanalysis, achieving efficient mapping between structural parameters (inputs) and structural responses (outputs) to enhance the reliability and application value of 3D printed material performance predictions.

**2) Figure 4 needs the scale bar.**

Answer: Thanks for pointing this out. We have updated Figure 4 by adding individual scale bars

**Figure 4** Display of Printed Components

**3)** **Figure 2 is not a professional image. Write all part on the image.**

Answer: Thanks for the suggestion. We have fully redrawn Figure 2 and placed all labels directly on the figure.

**Figure 2** Printing Equipment

**4) In keywords, "Parameter Identification" cannot be found in the abstract or the title. Then, how could it be a keyword?**

Answer: Thank you for the correction. We have revised the Keywords by replacing “Parameter Identification” with “Parameter Updating” to ensure consistency with the Title and Abstract. We also performed a consistency check across the manuscript to use “parameter updating” uniformly.

**5) For Figure 5, (a), (b), and (c) must be described in the title and not below the images. Consider this issue for all figures.**

Answer: Thank you for the guidance. We have revised Figure 5 so that the descriptions for panels (a), (b) are now included in the figure title line, rather than below the images.

(a) Experimental setup for modal testing

(b) FE model for specimen

Figure 5. Experimental setup and FE model for specimen

**6) The title of Figure 5 is very short, as mentioned before. Also, there is no descriptions for (a) and (b). Check also all other figures.**

Answer: Thank you for the reminder. As noted in our response to Comment 5, we have expanded the caption of Figure 5 to include an informative title. We also reviewed all other figures and applied the same standard.

**7) Where is the reference for Table 3? It must be mentioned in the title of the table.**

Answer:

Thanks for the attention to this point. In fact, Table 3 reports the flexural moduli identified for all members using the proposed method. The parameter θ denotes the updating factor, which is the relative change with respect to the initial value of 600 MPa. This initial value was estimated with reference to the full-infill modulus (4000 MPa) reported in [37]. We have cited [37] in the paragraph immediately following Fig. 8 in the revised manuscript.

**8) The discussion is not just to describe what happens. The results must be compared to other results of other published articles.**

Answer:

Thanks for the suggestion. In the revised manuscript, we have incorporated direct comparisons between our findings and results reported in previous studies to strengthen the discussion. Specifically:

1. **Comparison with literature [41] on elastic modulus:**
   In the discussion related to Figure 9, we added a comparison between the relative differences in elastic modulus observed for PLA and PETG at different infill ratios and the reported growth in bending elastic modulus from the literature. The revised text now reads:
   *“These observed relative differences are comparable to the 20–25% growth in bending elastic modulus reported in literature [41] through three-point bending tests under static loading when infill density increased from 50% to 75%.”*
2. **Comparison with literature [42] on frequency deviation:**
   In the discussion of frequency errors after model updating, we now compare the larger deviations observed for ASA at 30% infill with the magnitude of elastic modulus deviation reported in literature [42]. The revised text states:
   *“…which is very close to the magnitude of deviation in the elastic modulus identified in literature [42].”*

**9) It is not clear which references were added, not highlighted!**

Answer: Thanks for pointing this out. In the revised tracked manuscript we have now clearly marked all references changes:

Added references: marked in red in the Reference list.

Removed references: the original entries are highlighted in yellow in the tracked manuscript.

**10) As the second reviewer asked, SEM images must be added to the manuscript.**

Answer: Thanks for the suggestion. In response, we have added the scanning electron microscopy (SEM) images of the specimens along the printing height direction, which are now included in Figure 4 of the revised manuscript. For better logical consistency, the order of Figures 3 and 4 has been adjusted accordingly.

As described in the revised text:

“Figure 4 presents detailed views of the internal infill grid structures within the printed components. As shown in Figure 4(a) and (b), the specimens with 15% and 30% infill densities exhibit square grid patterns with dimensions of 3 mm and 6 mm, respectively, which are consistent with the grid configurations specified during printing. However, the component with 5% infill density in Figure 4(c) displays incomplete square patterns due to the constraints imposed by the low infill density setting. Figure 4(d), (e), and (f) provide scanning electron microscopy (SEM) images captured along the infill direction for the three components shown in Figure 4. These SEM images reveal that the actual layer height closely matches the preset printing layer thickness of approximately 0.2 mm across all specimens. The layer stacking appears relatively uniform throughout, with PETG material demonstrating the most consistent and orderly layering arrangement. These observations indicate that the printer’s process parameters were well-controlled during fabrication.”
