# Supplementary material for: Nominal elastic modulus assessment in 3D-printed components under varying printing parameters using Bayesian methods and random forest surrogate modeling
Source: PLoS One. 2025 Dec 5;20(12):e0338204. doi: 10.1371/journal.pone.0338204 (PMC12680227; doi:10.1371/journal.pone.0338204)
Supplement: Attachment — Submitted filename: 2025.11.13 plos one_2 review.docx [file pone.0338204.s006.docx]

**Additional Editor Comments:**

Still, it needs modifications as follows,

**1) The reference must be added just before the formulation in the main text. A general text that they are for [21-23], is not enough. Put the reference, just before the formulation, in the sentence before the formulation.**

**Response:** Thank you for the helpful suggestion. We have revised Section 2.1 (Bayesian method) so that each equation (Eqs. 1–3) and the MCMC step list are now introduced by sentences containing their corresponding, specific references immediately before the equations.

Specifically,

- The introductory sentence before Eq. (1) now cites [27], which supports the standard likelihood formulation for modal-data model updating.
- Eqs. (2) and (3) remain based on the same formulation under the Gaussian-error assumption.
- The sentence introducing the MCMC procedure now explicitly cites [29], which describes the Metropolis–Hastings sampling steps.

**2) Still, all figure titles are very short with not-enough descriptions. Moreover, the descriptions for (a) and (b) should be in the figure title and not under the images.**

**Response:** Thank you for the reviewer of this helpful suggestion. We have thoroughly revised all figure captions to make them self-contained and moved all panel descriptions from images into the captions.

**3)** **Figure 7 is not a finite element model! Please correct it.**

**Response:** Thank you for the observation. We agree that the previous Figure 7 was a schematic rather than an FE model. We have corrected the caption to: “Figure 7. Schematic of boundary conditions and sensor locations used for the beam.” All in-text references were updated accordingly. The actual finite-element mesh is shown and referenced in Fig. 5(b).

**4) Just two sentences are added for the discussion! It is not enough.**

**Response:** Thank you for the helpful comment. We have expanded the discussion in the two relevant places.

**After Table 3**, we added a paragraph explaining the material- and infill-dependent modulus gaps. The new text states that the trends are consistent with the monotonic increase of bending stiffness with relative density in FDM parts and with commonly reported material rankings; PLA typically exhibits a higher glassy modulus and stronger inter-road bonding than PETG and ASA [41]. It further explains that, within the 5–30% infill range, the effective modulus reflects both intrinsic polymer stiffness and cellular-solid scaling (reduced void fraction and shear-lag), and notes the practical implication that the observed 20–30% PLA–PETG difference at fixed infill can rival the gain from increasing infill from 15% to 30%, with only a modest mass increase.

**Before Figure 10**, we inserted a quantitative rationale for the small residual errors after updating. The revised text explains that, because natural frequency scales with the square root of the stiffness-to-mass ratio, a 4–6% spread in elastic modulus commonly observed for printed coupons leads to an expected ≈2–3% spread in frequency at fixed mass [42]. We also note ~1% additional, mode-dependent shifts due to slight clamp variability and the 12 g sensor’s added mass/compliance, and we point out that MAC values >0.98 (typically ≈0.99) indicate that the remaining gap is mainly a slight stiffness-amplitude bias rather than a mode-shape mismatch.

**5) Still, it is not clear what is the reference for Table 3. If they are from a reference, the number of reference must be added to the title. If not, it is not clear how these data were obtained. If tested, more details must be provided.**

**Response:** Thank you for pointing this out. We have clarified the provenance of Table 3 in the text and in the table caption. The values in Table 3 are identified in this work from our experimental modal measurements using the Bayesian updating procedure in Section 2.1; they are not taken from the literature. The nominal modulus $E_{0}=600$MPa is an assumed initial baseline used to non-dimensionalize $\theta$ and to initialize the FE model and is not reported as a result. We have added explicit statements to the paragraph introducing Table 3 and revised the table caption accordingly (see Table 3).
