# Supplementary material for: Nominal elastic modulus assessment in 3D-printed components under varying printing parameters using Bayesian methods and random forest surrogate modeling
Source: PLoS One. 2025 Dec 5;20(12):e0338204. doi: 10.1371/journal.pone.0338204 (PMC12680227; doi:10.1371/journal.pone.0338204)
Supplement: Attachment — Submitted filename: 2025.11.17 plos one_2 review.docx [file pone.0338204.s007.docx]

**Additional Editor Comments:**

Almost done; however, still there are several formulations without references. Add them in the proof stage.

**Response to the Editor:**

We sincerely thanks editor for the careful reading and the helpful comment that “Almost done; however, still there are several formulations without references. Add them in the proof stage.”

In the revised manuscript, we have rechecked the entire text and added explicit references to support previously uncited formulations:

(i) additional citations to recent works on mechanical characterization of FDM-printed polymers have been included in the Introduction (Ji et al. [18], Gallup et al. [19], and Mencarelli et al. [20]);

(ii) the discussion of model updating in civil and aerospace structures is now supported by the Bayesian FE model updating literature [21–23];

(iii) textbook-style descriptions of the Random Forest algorithm and the Latin Hypercube Sampling design have been complemented with appropriate references [24,25,43].

These changes will also be carefully checked again at the proof stage to ensure that all formulations that rely on prior work are properly referenced. We appreciate the editor’s positive evaluation of our manuscript and the constructive reminder.
